# Expectations of the Physiological Responses Can Change the Somatosensory Experience for Acupuncture Stimulation

**DOI:** 10.3389/fnins.2019.00074

**Published:** 2019-02-12

**Authors:** Hyun-Seo Song, Won-Mo Jung, Ye-Seul Lee, Seung-Woo Yoo, Younbyoung Chae

**Affiliations:** ^1^Acupuncture and Meridian Science Research Center, College of Korean Medicine, Kyung Hee University, Seoul, South Korea; ^2^Department of Anatomy and Acupoint, College of Korean Medicine, Gachon University, Seongnam, South Korea

**Keywords:** acupuncture, biosignal, *de qi*, expectation, psychophysics

## Abstract

**Objective:** Humans interpret sensory inputs based on actual stimuli and expectations of the stimuli. We investigated whether manipulating information related to the physiological response could change the somatosensory experience of acupuncture.

**Methods:** Twenty-four participants received tactile stimulations with a von Frey filament on the left arm. Participants were informed that they would receive acupuncture stimulations at different angles while they were presented with changes in their peripheral blood flow (PBF) measured with Laser Doppler perfusion imaging. However, in reality, they were observing premade pseudo-biosignal images (six sessions: one circular, two rectangular elongated, two diagonally elongated, and one cross-fixation [control] shape). After each session, the participants reported the intensity and location of the *de qi* sensations perceived on their arm using a bodily sensation mapping tool. The spatial patterns of the somatic sensations were visualized using statistical parametric mapping. The F1 score was calculated to measure the similarity between the presented pseudo-biosignals and reported *de qi* response images.

**Results:** The spatial configurations of the presented pseudo-biosignal images and *de qi* response images were similar. The rectangular elongated pseudo-biosignal shape had a significantly higher F1 score compared to the control. All tactile stimulations produced similar levels of enhanced PBF regardless of the pseudo-biosignal shape.

**Conclusion:** The spatial configurations of somatic sensations changed according to the presented pseudo-biosignal shape, suggesting that expectations of the physiological response to acupuncture stimulation can influence the perceived somatic sensation.

## Introduction

A fundamental characteristic of acupuncture treatment is the elicitation of unique somatic sensations around points (i.e., *de qi* sensations) stimulated with the insertion of a needle (Chae et al., 2013; Chae and Olausson, 2017). Achieving an appropriate *de qi* sensation is believed to be a key component of acupuncture treatment (Kong et al., 2005; Mao et al., 2007). In a clinical research data-mining study, 82.1% of studies supported that *de qi* sensations were closely related to clinical efficacy (Pan et al., 2017). *De qi* sensations include a combination of various sensations, such as heaviness, numbness, soreness, distention, and even a spreading sensation far from the stimulus site (MacPherson and Asghar, 2006). Recently, spatial patterns of acupuncture-induced sensations, including sensations propagated along the acupuncture meridians, have been demonstrated based on a geographic information system using a bodily sensation map (BSM) (Beissner and Marzolff, 2012; Jung et al., 2016). The BSM, one of a digital pen-and-paper platform, is useful to measure location and intensity of the bodily sensation (Jung et al., 2017a,c).

Humans perceive somatic sensations by integrating afferent signals and higher cognitive processes. For example, placebo stimulations have been reported to cause considerable and widespread sensations despite a lack of peripheral stimulus (Beissner et al., 2015). Moreover, in a clinical trial using laser acupuncture, remarkable *de qi* sensations were elicited without any cutaneous sensory input (Salih et al., 2010). Furthermore, brain activation associated with acupuncture stimulation was influenced by enhanced bodily awareness and bodily attention around the acupoint (Chae et al., 2015; Jung et al., 2016). In a recent study, expectations of acupuncture stimulation elicited a distinct somatic sensation experience and activation of the salience network in the brain, even without an afferent somatosensory signal (Jung et al., 2018). Together, these results suggest that experiences and expectations prior to tactile stimulation can influence the perception of somatic sensations.

These properties of somatic sensory experience suggest that *de qi* sensation patterns can be influenced by expectations of psychophysiological responses to acupuncture stimulations. In this study, we hypothesized that biosignal information (i.e., peripheral blood flow change) presented as a psychophysiological response to acupuncture would alter the spatial configurations of somatic sensations in response to tactile stimulations. More specifically, since propagated sensations induced by acupuncture were prominently along perpendicular and horizontal lines on the body, it can be assumed that rectangular elongated pseudo-biosignal of acupuncture can efficiently influence on the perceived somatic sensations. When tactile stimulations were applied to the arm, the perceived sensation showed circular-shaped spatial patterns. Thus, it is assumed that there will be no differences in the spatial patterns of the *de qi* sensations between the circular images session and the control session.

To test this hypothesis, we presented participants with different Laser Doppler perfusion images of pseudo-biosignal shapes mimicking changes in peripheral blood flow (PBF) and compared the spatial configurations of the somatic sensations induced by tactile stimulation.

## Materials and Methods

### Subjects

We recruited 24 healthy volunteers via advertisements targeting students of Kyung Hee University and Korea University. None of the participants of this study had any history of cardiac, neurological, psychiatric, or visual disorders. Participants refrained from drinking alcohol or caffeine or from taking any drugs or medications for 12 h before the experiment. All participants received a detailed explanation of the experiment and provided written informed consent. This experiment was conducted in accordance with the Declaration of Helsinki and approved by the Institutional Review Board of Kyung Hee University. All procedures were conducted in a quiet and temperature-controlled room (24 ± 2°C) throughout the experiment.

### Experimental Design and Procedure

Each participant was asked to sit in a comfortable position in front of a computer monitor before the experiment. Participants were informed that they would receive acupuncture stimulation at various angles and that they would be presented with changes in their PBF measured using Laser Doppler perfusion imaging. However, in reality, the participants were presented with premade pseudo-biosignal images (one circular, two rectangular elongated, two diagonally elongated, and one cross-fixation [control] shape). A total of six sessions were conducted and all participants were unaware of the procedures. The pseudo-biosignals and control image were presented in a pseudorandom order. The experiment followed a within-subject crossover design, in which the independent variable was the pseudo-biosignal shape. All participants were unaware of the procedures. The pseudo-biosignals were presented in a pseudorandom order.

**FIGURE 1:**
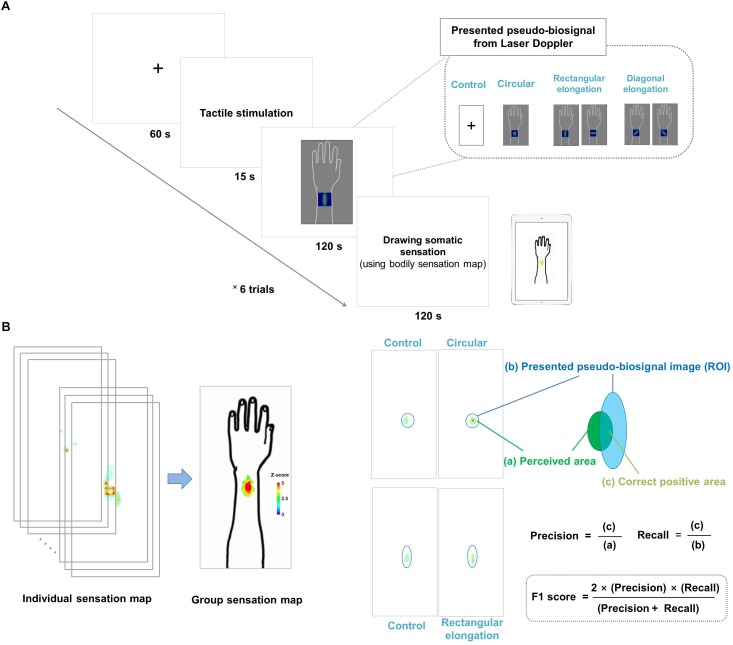
**(A)** Experimental design. All participants were shown images of four different pseudo-biosignal shapes. Each participant received a tactile stimulation at the same site on the left arm using a von Frey filament. In each session, the participants were asked to report the location and intensity of the resulting sensation on a bodily sensation map tool. **(B)** Data visualization and data analysis. The spatial distributions of the somatic sensations were visualized using the human body template by applying *Z* scores. The F1 score was used to measure the similarity between the presented pseudo-biosignal image and reported somatic sensation area. The F1 score is calculated as (2 × precision × recall)/(precision + recall), where precision is the area of the correct positive results divided by the area of all relevant samples from the perceived *de qi* sensation area (c/a) and recall is the area of correct positive results divided by the area of all positive results from the region of interest of the presented pseudo-biosignal image (c/b).

Each experimental session lasted for 315 s, during which the participants looked at the cross fixation for the preparation period for 60 s and they received tactile stimulation for 15 s. Right after stimulation, participants were presented with the pseudo-biosignal for 120 s. They were required to fix their gaze on the computer monitor to view the change of the pseudo-biosignal immediately following the tactile stimulation. The actual PBF around the stimulation site was measured before and after stimulation. Finally, participants were asked to mark the areas of induced sensation on a BSM for 120 s. A 2-min break was given between sessions to allow the participants to relax (Figure 1A).

Basic demographic data were collected from all participants before commencing the experiments. Participants also evaluated the expectancy and fear of the acupuncture treatment using the Acupuncture Expectancy Scale (AES) and Acupuncture Fear Scale (AFS) (Kim et al., 2013, 2014).

### Tactile Stimulation

Each participant received a tactile stimulation at the same site of the left arm (TE5 acupoint: on the posterior aspect of the forearm, at the midpoint of the interosseous space between the radius and ulna, 2 B-cun proximal to the dorsal wrist crease). All tactile stimulations were delivered using a 60-g von Frey filament (North Coast Medical Inc., San Jose, CA, United States). Tactile stimulations designed to mimic acupuncture stimulation were given at a frequency of 1 Hz for 15 s. The stimulated sites on the arm were hidden from the participants’ sight to make them believe that they were receiving real acupuncture stimulations. All participants were told that they would receive different acupuncture stimulations at various angles in each session. However, we did not inform the participants which angle of the needle was being inserted. In acupuncture practice, it is common to change the angle of needle insertion according to the site and the disease. Thus, participants might have supposed that various directions of the pseudo-biosignal image are derived from various angle of the needle stimulation.

### Measurement of Perceived Tactile Sensations Using the Bodily Sensation Map

Following stimulation, the participants were asked to report the locations and intensity of bodily sensations using the BSM^1^. This tool presents a template of the human body as two-dimensional images, including a lateral view of the left arm. The perceived sensations after each session were recorded in 1,024 × 512 matrices on an iPad (Apple Inc., Cupertino, CA, United States). Participants could express the intensity of the somatic sensation by changing the color of the points on a continuous color map via successive strokes with a touch pen (Adonit Inc., Austin, TX, United States).

### Measurement of Peripheral Blood Flow Using Laser Doppler Perfusion Imaging

Measurements of peripheral blood perfusion around the stimulation site were performed using a Laser Doppler perfusion imager (PeriScan PIM 3 System; Perimed AB, Järfälla, Sweden). The left arm of the participants was stabilized with a kapok-filled vacuum cushion to maintain its position, and measurements were conducted every 10 s for 180 s before and after stimulation. PBF (symbolized as R*i*, [*i* = 1, 2, 3, …, 18]) was calculated as the average perfusion value during each 10-s scan in the region of interest (ROI) (a 2 cm × 2 cm square centered on the TE5 acupoint), and the change in PBF was defined as ΔPBF = (R*i* - R1)/R1. Each 10 s PBF was subtracted and divided by a baseline (collected over 30 s) in each session, which yielded the change in scores reflecting the increase in PBF from the baseline.

### Data Analysis

To determine the spatial patterns of the sensations under each condition, we extracted parametric maps of bodily sensation using the BSM tool. Individual datasets for each subject were normalized within the range of 0–1. The normalized BSMs were subjected to group-level analysis of the statistical parametric maps. The group-level analysis consisted of a random effects analysis using the pixel-wise univariate *t*-test of individual BSMs for each session (3dttest++, AFNI^2^). False discovery rate (FDR) corrections were carried out to account for false positives due to multiple comparisons (FDR-corrected *p* < 0.05). The resulting *t-*value maps were transformed into *Z* scores that reflected significant spatial information of the bodily sensation to tactile stimulation. The information was color-coded based on the *Z* score.

The regions of color changes for each pseudo-biosignal image were extracted as the ROI of each condition. A larger intersection area and smaller complementary area between the ROI and reported somatic sensation area indicated a greater similarity between them. We used the F1 score to evaluate the similarity between the presented pseudo-biosignal images and reported areas of somatic sensation. The F1 score is generally used as a measure of a test’s accuracy in binary classifications (Lipton et al., 2014), and is the harmonic mean of precision and recall, calculated as (2 × precision × recall)/(precision + recall). Here, precision is defined as the area of correct positive results divided by the area of all positive results (from the area of the reported sensation), and recall is the area of correct positive results divided by the area of all relevant samples (from the ROI of the presented pseudo-biosignal image). In this study, precision was the fraction of the intersection area of the reported sensation area and recall was the fraction of the intersection area of the ROI of PBF changes for a given pseudo-biosignal image. The F1 score was calculated for each participant and pseudo-biosignal type (i.e., circular, rectangular elongated, and diagonally elongated shapes) (Figure 1B).

Statistical analyses of the F1 score and PBF data were performed using the R software package^3^. The F1 score for each pseudo-biosignal type was compared to the F1 score extracted from the control session with the same ROI. Paired *t*-test was used to identify significant differences. We conducted repeated measures analysis of variance (ANOVA) to compare the intensities of the change in PBF under each condition over time to determine the effect of time and biosignal type (i.e., the four conditions).

## Results

### Baseline Characteristics

This study was carried out in 24 right-handed participants (aged 18–37 years; mean = 23.4, standard deviation = 4.0; 13 females). The AES score was 12.6 ± 0.6 and the AFS score was 29.0 ± 2.2.

### Effect of Expectation on Tactile Stimulation Sensory Experience

**FIGURE 2:**
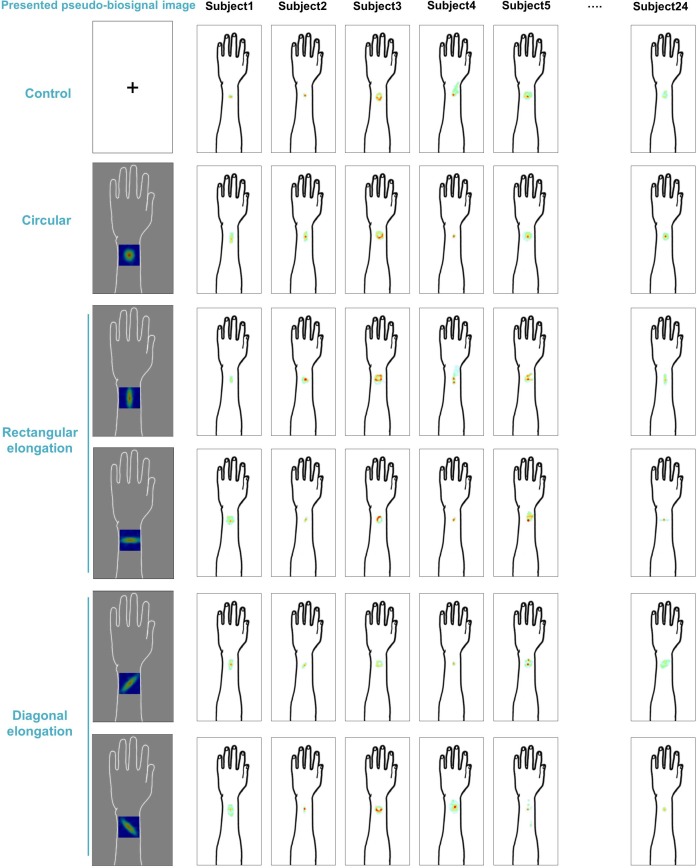
Individual map for somatic sensation spatial patterns following tactile stimulation in each condition. Six examples of individual *de qi* sensation drawings.

**FIGURE 3:**
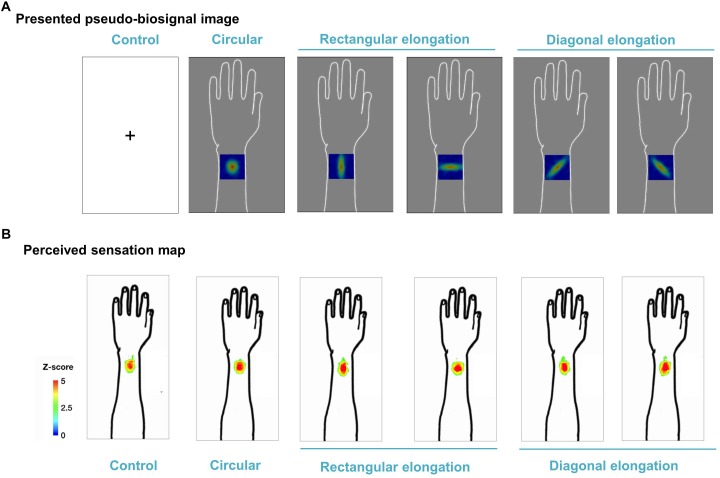
Group map for somatic sensation spatial patterns following tactile stimulation in each condition. **(A)** Presented pseudo-biosignal images (one circular, two rectangular elongated, two diagonally elongated, one cross-fixation [control] shape). **(B)** Group sensation map. The bodily sensation maps were similar to the presented pseudo-biosignal images and *de qi* response images for the rectangular elongated shape. The group-level analysis consisted of a random effects analysis using a pixel-wise univariate *t*-test of individual sensation maps for each session (FDR-corrected *p* < 0.05). The color coding represents the *Z* score.

We demonstrated individual spatial patterns of somatic sensation following tactile stimulation in each condition (Figure 2). After group level analysis, we found that the spatial configurations of the presented pseudo-biosignal images and the *de qi* response images were similar in the rectangular elongated shape condition, suggesting that presentation with different pseudo-biosignal shapes could influence the spatial configuration of the somatic sensation to the same tactile stimulation (Figure 3).

**FIGURE 4:**
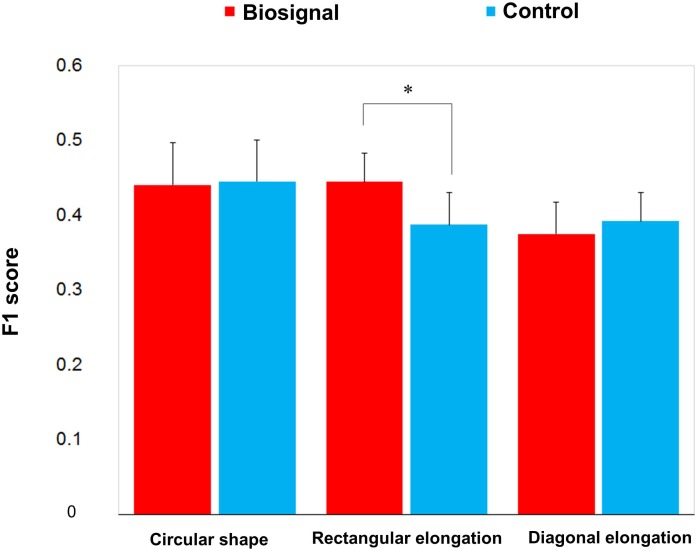
Similarity between the presented biosignal images and *de qi* response images. The F1 score indicates the similarity between the presented pseudo-biosignal image and reported somatic sensation area. Presentation with the rectangular elongated-shaped pseudo-biosignal resulted in a greater similarity between the presented biosignal image and *de qi* response pattern compared to the control. The red bars represent the similarity with the pseudo-biosignal images and the blue bars represent the similarity without presentation of pseudo-biosignal images (control). A paired *t*-test was conducted to identify significant differences between the pseudo-biosignal image and control conditions. ^∗^represents *p* < 0.05.

F1 score was used to evaluate the similarity between the presented pseudo-biosignal images and reported areas of somatic sensation. Based on the F1 score, the association between the rectangular elongated pseudo-biosignal image and resulting *de qi* response image exhibited a significant similarity compared to control (0.445 ± 0.039 vs. 0.388 ± 0.043, *t* = 2.168, *p* < 0.05). By contrast, the circular and diagonally elongated pseudo-biosignal images and *de qi* response images did not exhibit any significant similarity (Figure 4). These findings suggest that the rectangular elongated pseudo-biosignal image, but not other conditions, can enhance similarity between the pseudo-biosignal images and perceived somatosensation.

### Peripheral Blood Flow Response to Tactile Stimulation

**FIGURE 5:**
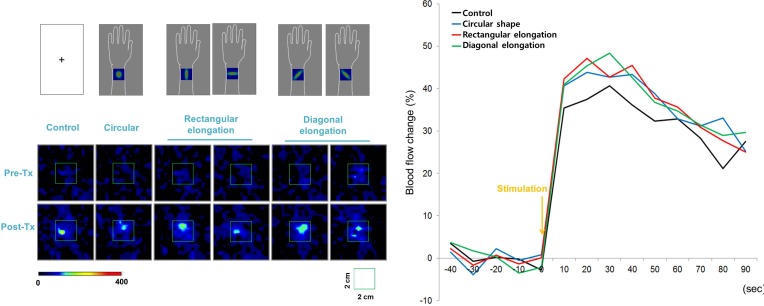
Peripheral blood flow (PBF) response to tactile stimulation. All tactile stimulations produced, similarly, enhanced intensities of PBF around the acupoints across the four pseudo-biosignal shape conditions.

All tactile stimulations produced similar levels of enhanced PBF around the acupoint regardless of the pseudo-biosignal shape, and participants exhibited a 40% increase in PBF within 30 s under all conditions (Figure 5). Repeated measures ANOVA revealed a significant effect of time [*F*_(3,13)_ = 14.877, *p* < 0.001]; however, the interactive effect of biosignal type × time [*F*_(3,39)_ = 0.386, *p* > 1.000] and biosignal type [*F*_(3,3)_ = 0.193, *p* > 0.901] showed no significant effects.

## Discussion

The purpose of this study was to determine whether the spatial configurations of *de qi* sensations could be influenced by manipulating the shape of pseudo-biosignal images presented to participants. The results revealed similarities between the spatial configurations of the presented pseudo-biosignal images and somatic sensation response patterns. In particular, presentation with the rectangular elongated pseudo-biosignal resulted in a similar somatic sensation spatial configuration. These findings suggest that biosignal information of psychophysiological responses to acupuncture can change somatic sensation spatial configurations. Overall, this study provides evidence that information from prior experiences and expectations can influence the interpretation of sensory inputs.

Somatic sensations of acupuncture stimulations can be established not only by afferent signals, but also by higher cognitive process, such as expectations. We previously found that expectations of acupuncture stimulations could trigger considerable *de qi* sensations and brain activations in the salience network, even in the absence of actual afferent signals (Jung et al., 2015, 2018). The main finding of the present study is that the spatial configurations of sensory experiences can be altered by biosignal information. To test this, we presented participants with different pseudo-biosignal shapes mimicking Laser Doppler perfusion images to manipulate their expectations of the physiological changes to acupuncture stimulation. Even though we did not explicitly explain the associations between the pseudo-biosignal shapes and *de qi* sensation patterns, different expectations of physiological changes to acupuncture stimulation successfully induced different somatic sensation spatial configurations. Recent findings in cognitive neuroscience suggest that the brain can actively make inferences based on prior experiences and expectations (Buchel et al., 2014). In other words, information from prior experiences can be used to generate expectations about future perception and interpret sensory inputs (Wiech, 2016). Therefore, we assume that the altered sensory experiences in this experiment might have been derived from the implicit expectation of the association between the physiological response to acupuncture stimulations and the experience of somatic sensations.

When somatic sensations are induced by an acupuncture needle, *de qi* sensations and brain activations to acupuncture stimulation can vary among sessions and participants (Kong et al., 2007; Napadow et al., 2009). Therefore, to control the intensity of the tactile stimulation and induce the same intensity of somatic sensations among tests, we applied a von Frey filament to the TE5 acupoint in the left arm. This method can control for both the tactile stimulation of the cutaneous somatosensory receptor at the acupuncture point and the cognitive processing of participants expecting an acupuncture stimulation (Napadow et al., 2013; Chae et al., 2015). The stimulated sites on the arm were hidden from sight and all participants believed that they received acupuncture stimulations. Moreover, this experiment showed that all tactile stimulations produced, similarly, enhanced PBF around the acupoint regardless of the presented pseudo-biosignal shape, supporting that alterations of spatial patterns to tactile stimulation in rectangular elongated shape condition were not induced by differences in tactile stimulation evoked physiological responses.

The spatial configurations of the presented pseudo-biosignal images and *de qi* response images were similar, especially in the case of the rectangular elongated shape. Meanwhile, the circular pseudo-biosignal shape was created based on preliminary data of the spatial configurations of *de qi* sensations to tactile stimulations. When tactile stimulations were applied to the left arm, the *de qi* sensation elicited circular-shaped spatial configurations around the stimulation site. As expected, there were no differences in the spatial configurations of the *de qi* sensations between the session with the circular pseudo-biosignal image and the control session without pseudo-biosignal images. Meanwhile, the participant somatic sensation pattern responses to tactile stimulation were highly similar to the presented rectangular elongated (but not diagonally elongated) pseudo-biosignal pattern. In this experiment, the participants expected that they would receive acupuncture treatment at various angles. Interestingly, propagated sensations induced by acupuncture have been reported along perpendicular and horizontal lines on the body (Beissner and Marzolff, 2012; Jung et al., 2016). Since diagonal spatial patterns of *de qi* sensations are unusual, the participants may have had weaker expectations of diagonally elongated-shaped response perception patterns.

Perceived sensation to acupuncture stimuli, like other pain perceptions, can be influenced by prior knowledge or expectations (Buchel et al., 2014; Jung et al., 2017b; Ongaro and Kaptchuk, 2019). In this study, we manipulated participants’ expectation by using pseudo-biosignal images mimicking the changes in the blood flow during Laser Doppler perfusion imaging. We did not tell them any association between the biosignal from blood flow change and perceive sensations. However, the spatial patterns of *de qi* sensation in response to tactile stimulation were influenced by the information, especially in more plausible response condition. These findings suggest that the implicit expectations from the physiological changes from their own body can have influence on the perception of the tactile stimulation. From this study, the prospective studies include manipulating expectation by alterations of biosignals from the body can be applied to the modulations of other somatosensation areas such as pain control.

In summary, this study reveals that the spatial configurations of *de qi* sensations in response to tactile stimulation can be influenced with visual biosignal information. This suggests that information on physiological responses to acupuncture stimulations can change participants’ expectations of the perception of somatic sensation and interpretation of the stimulations. In future studies, it will be necessary to further clarify the characteristics of the cognitive factors in the perception of acupuncture stimulations.

## Author Contributions

H-SS, Y-SL, and YC conceived and designed the experiments. H-SS and S-WY performed the trial. H-SS, W-MJ, and YC analyzed the data. H-SS, W-MJ, and YC discussed the data. H-SS and YC wrote the first draft of the paper. H-SS, W-MJ, Y-SL, S-WY, and YC revised the paper and approved the final version.

## Conflict of Interest Statement

The authors declare that the research was conducted in the absence of any commercial or financial relationships that could be construed as a potential conflict of interest. The handling Editor is currently editing a Research Topic with one of the authors YC, and confirms the absence of any other collaboration.

## Footnotes

1 http://cmslab.khu.ac.kr/downloads/bsm

2 http://afni.nimh.nih.gov/afni

3 http://cran.r-project.org
